# Optimal implementation of the 2019 ESC/EAS dyslipidaemia guidelines in patients with and without atherosclerotic cardiovascular disease across Europe: a simulation based on the DA VINCI study

**DOI:** 10.1016/j.lanepe.2023.100665

**Published:** 2023-06-08

**Authors:** Julia Brandts, Sarah Bray, Guillermo Villa, Alberico L. Catapano, Neil R. Poulter, Antonio J. Vallejo-Vaz, Kausik K. Ray, DA VINCI study group

**Affiliations:** a Department of Primary Care and Public Health, Imperial Centre for Cardiovascular Disease Prevention, School of Public Health, Imperial College London, London, UK; b Department of Internal Medicine, University Hospital RWTH Aachen, Aachen, Germany; c Global Biostatistical Science, Amgen Ltd, Cambridge, UK; d Health Economics & Outcomes Research, Amgen (Europe) GmbH, Risch-Rotkreuz, Switzerland; e IRCCS MultiMedica, Milan, Italy; f Department of Pharmacological and Biomolecular Sciences, University of Milan, Milan, Italy; g Imperial Clinical Trials Unit, Imperial College London, London, UK; h Department of Medicine, Faculty of Medicine, University of Seville, Seville, Spain; i Clinical Epidemiology and Vascular Risk, Institute of Biomedicine of Seville (IBiS), IBiS/Hospital Universitario Virgen del Rocío/University of Seville/CSIC, Seville, Spain

**Keywords:** Atherosclerotic cardiovascular disease, LDL-C, Lipid-lowering, ESC/EAS guidelines, Cardiovascular risk

## Abstract

**Background:**

The impact of the stepwise implementation of the 2019 European Society of Cardiology (ESC)/European Atherosclerosis Society (EAS) treatment algorithm on low-density lipoprotein cholesterol (LDL-C) goal attainment was simulated in patients from the DA VINCI study.

**Methods:**

Monte Carlo simulation was used to evaluate treatment optimisation scenarios, based on a patient's risk category: statin intensification (step 1), addition of ezetimibe (step 2), and addition of a proprotein convertase subtilisin/kexin type 9 (PCSK9) inhibitor (step 3). Residual cardiovascular risk and predicted relative and absolute risk reduction (RRR and ARR) in cardiovascular events were assessed.

**Findings:**

In DA VINCI, 2482 patients did not achieve their 2019 ESC/EAS LDL-C goals and were included in the simulation. In patients without atherosclerotic cardiovascular disease (ASCVD) (*n* = 962), 27.0% (*n* = 259) and 57.0% (*n* = 548*)* are likely to achieve their LDL-C goals at step 1 and step 2, respectively. Of those at very high risk without ASCVD (*n* = 74), 88.1% (*n* = 65) are likely to achieve their LDL-C goals at step 3. In patients with ASCVD (*n* = 1520), 12.0% (*n* = 183), 42.1% (*n* = 641) and 93.2% (*n* = 1416) are likely to achieve their LDL-C goals at steps 1, 2 and 3, respectively. In patients with and without ASCVD, treatment optimisation may result in mean simulated RRR of 24.0% and 17.7%, respectively, and ARR of 8.1% and 2.6%, respectively.

**Interpretation:**

Most patients at high cardiovascular risk are unlikely to achieve LDL-C goals through statin optimisation and ezetimibe, and will require a PCSK9 inhibitor, leading to greater reduction in cardiovascular risk.

**Funding:**

10.13039/100002429Amgen.

**Research in context**

**Evidence before this study**

The 2019 European Society of Cardiology (ESC)/European Atherosclerosis Society (EAS) guidelines for the management of dyslipidaemia recommend lipid-lowering therapy (LLT) to reduce low-density lipoprotein cholesterol (LDL-C) levels. Recent evidence has suggested that combination therapy, such as statins and ezetimibe, or the addition of a proprotein convertase subtilisin/kexin type 9 (PCSK9) inhibitor, is required for patients to achieve their ESC/EAS LDL-C goals. We searched PubMed on 14 October 2022 using the search terms “dyslipidaemia” AND “guidelines” AND “simulation”, with no restriction on language. Thirty-three studies were identified. Published studies also highlight that treatment intensification would be required for patients to achieve their LDL-C goals. In a simulation study of 25,466 adults with a recent myocardial infarction from the SWEDEHEART registry, it was demonstrated that optimisation of the 2019 ESC/EAS LLT algorithm may lead to goal attainment in 90% of patients. In addition, published studies indicate that gaps exist between guideline recommendations and current practice regarding LLT use.

**Added value of this study**

The cross-sectional, observational DA VINCI study examined LLT use in primary and secondary care, across 18 countries in Europe, for the primary and secondary prevention of ASCVD. In this analysis, a simulation was used to examine the impact of the stepwise implementation of the 2019 ESC/EAS treatment algorithm on LDL-C goal attainment in patients from DA VINCI who did not achieve their LDL-C goals. Residual risk and relative and absolute risk reduction (RRR and ARR) were also assessed after treatment optimisation. This analysis extends and builds on the findings from the SWEDEHEART registry to a broader European population and also includes data on simulated CV risk. Results from this study suggest that over half of patients at high risk without ASCVD would attain their 2019 ESC/EAS risk-based LDL-C goals by optimising their statins therapy and adding ezetimibe. However, for patients at very high risk, including those with atherosclerotic cardiovascular disease (ASCVD), the use of the combination therapy of statins, ezetimibe and a PCSK9 inhibitor would result in approximately 90% of patients achieving their risk-based LDL-C goals.

**Implications of all the available evidence**

In this analysis of patients from DA VINCI who did not achieve their LDL-C goals, optimisation of statin monotherapy alone would be insufficient to achieve the 2019 ESC/EAS risk-based LDL-C goals. The stepwise addition of ezetimibe is likely to result in at least twice as many patients achieving their LDL-C goals across all risk categories, compared with statin optimisation alone. Most patients at very high risk may require not only the optimisation of statins, but also the addition of both ezetimibe and a PCSK9 inhibitor to attain their risk-based LDL-C goals. For patients with ASCVD, if LLT was optimised as per the 2019 ESC/EAS algorithm, the estimated RRR and ARR of a cardiovascular (CV) event would be 24.0% and 8.1%, respectively, over 10 years. These results highlight the importance of optimising LLT to mitigate future CV events.

## Introduction

The 2019 European Society of Cardiology (ESC)/European Atherosclerosis Society (EAS) guidelines for the management of dyslipidaemia recommend lipid-lowering therapy (LLT) to reduce low-density lipoprotein cholesterol (LDL-C) levels, advocating a ‘lower is better’ approach for those at the highest risk.^1^ The reduction in LDL-C levels through the use of LLT is a key strategy to decrease the risk of cardiovascular (CV) events.^2^ LLT can be prescribed as a monotherapy or as a combination therapy, with statins and ezetimibe, or the addition of a proprotein convertase subtilisin/kexin type 9 (PCSK9) inhibitor. The 2019 ESC/EAS guidelines propose the addition of ezetimibe in patients who do not achieve their CV risk-based LDL-C goals with the maximum tolerated dose of statins as a first step.^1^ In patients who do not achieve their risk-based LDL-C goals with the maximum tolerated dose of statins and ezetimibe, the guidelines recommend the addition of a PCSK9 inhibitor for patients with atherosclerotic cardiovascular disease (ASCVD) and for those without ASCVD who are considered to be at very high CV risk or have familial hypercholesterolaemia (FH).

The aim of the cross-sectional, observational DA VINCI study was to describe LLT use in primary and secondary care, across 18 countries in Europe.^3^ The DA VINCI study demonstrated that the use of LLT to reduce CV risk consisted mostly of statin monotherapy (83%). In addition, only 9% of patients were receiving combination therapy of moderate- or high-intensity statins and ezetimibe, and only 1% were receiving moderate- or high-intensity statins and/or ezetimibe with a PCSK9 inhibitor. Overall, only 33% of patients included in the DA VINCI study achieved their risk-based LDL-C goals based on the 2019 ESC/EAS guidelines,^1^ and only 18% of patients considered to be at very high CV risk achieved their risk-based LDL-C goals.^3^ Indeed, multiple real-world studies have demonstrated that ESC/EAS goal attainment is sub-optimal for patients at very high CV risk. 4^4^^,^^5^ Thus, combination therapy, such as statins and ezetimibe, or the addition of a PCSK9 inhibitor, is likely required for patients to achieve their ESC/EAS LDL-C goals. Notably, two studies support the need for treatment intensification for patients with established coronary artery disease to achieve their ESC/EAS LDL-C goals.^6^^,^^7^ In a simulation study of 25,466 adults with a recent myocardial infarction from the SWEDEHEART registry, it was demonstrated that optimisation of the 2019 ESC/EAS LLT algorithm may lead to goal attainment in 90% of patients.^6^ In a similar study in 27,443 patients with coronary artery bypass surgery over 95% of patients achieved their LDL-C goals after LLT intensification.^7^

In this analysis of patients from DA VINCI who did not achieve their LDL-C goals, we aimed to simulate the impact on risk-based LDL-C goal attainment across CV risk categories through stepwise implementation of the treatment algorithm provided in the 2019 ESC/EAS dyslipidaemia guidelines. We also assessed potential residual CV risk and potential relative and absolute CV risk reduction (RRR and ARR) after treatment optimisation in patients from the DA VINCI study. Baseline risk was assessed using the REduction of Atherothrombosis for Continued Health (REACH) equation and the Systematic COronary Risk Evaluation (SCORE) in patients with and without ASCVD, respectively. This analysis extends and builds on previous studies' findings and includes a broader European population, patients with and without ASCVD (including those with coronary artery disease [CAD], peripheral arterty disease [PAD] and cerebrovascular disease [CeVD]), and data on simulated CV risk.

## Methods

### Study design

Details of the cross-sectional DA VINCI study have been published previously.^3^ In brief, 5888 adults (3000 without ASCVD and 2888 with ASCVD) prescribed LLT between June 2017 and November 2018, across 18 countries in Europe, were enrolled. Patients were eligible to participate if they were aged 18 years or older and had been prescribed LLT at enrolment or in the 12 months before enrolment and had an LDL-C measurement recorded in the 14 months before enrolment. All relevant data were obtained during a single routine clinic visit. Site selection and site capped enrolment facilitated the inclusion of patients in primary and secondary prevention of ASCVD in a 1:1 manner. Those with ASCVD were enrolled in a 1:2:2 ratio for CAD, PAD, and CeVD, respectively. The protocol for the DA VINCI study was registered at the European Union electronic Register of Post-Authorisation Studies (registration no. EU PAS 22075) and approved by the regional ethics committees or institutional review board from each site.

### Simulating the risk-based 2019 ESC/EAS treatment algorithm

For this analysis, patients were eligible for inclusion if they met all of the following inclusion criteria: (1) they were included in the primary analysis set in the DA VINCI study and were on a stable LLT regimen when their LDL-C measurement was taken; (2) they did not achieve their 2019 ESC/EAS risk-based LDL-C goals in the DA VINCI study; and (3) they were stabilised on a known intensity of statin and/or ezetimibe in the DA VINCI study. Patients who were already receiving a PCSK9 inhibitor were excluded from this analysis. Further information regarding the patients who were excluded from this analysis are included in the Supplementary Material. Stable LLT was defined as no change in dose or regimen of LLT for at least 28 days before the LDL-C measurement was taken. Patients were categorised as either without or with ASCVD. Patients categorised as without ASCVD were subsequently grouped into their respective CV risk category, which was assigned using SCORE. Patients with ASCVD were categorised as either recurrent or not recurrent, based on whether they had experienced at least two events within the preceding 2 years.

**Fig. 1:**
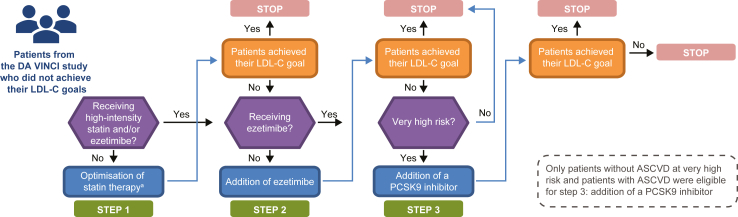
Treatment optimisation simulation. ^a^Uptitration of statins for patients not already receiving the highest available dose of their currently prescribed statin and not already receiving ezetimibe. The black horizontal arrows signify the patients who already are receiving high intensity statin at step 1 or those already receiving ezetimibe at step 2. These patients do not require further optimisation of their statin therapy (step 1) or addition of ezetimibe (step 2). ASCVD, atherosclerotic cardiovascular disease; LDL-C, low-density lipoprotein cholesterol; PCSK9, proprotein convertase subtilisin/kexin type 9.

The Monte Carlo method was used to simulate treatment optimisation scenarios with 1000 simulations generated to achieve the 2019 ESC/EAS risk-based LDL-C goals, based on published efficacy estimates (Supplementary Table S1).^8^^,^^9^ The Monte Carlo method is useful for simulating outcomes in different scenarios using repeated random sampling. Patients followed a treatment optimisation algorithm based on their risk category, progressing to the next step if they did not achieve their risk-based LDL-C goals in the previous step (Fig. 1). In DA VINCI, risk categories for individuals defined as primary prevention were assigned using SCORE and patients were categorised as having a low, moderate, high or very high CV risk according to the 2019 ESC/EAS guidelines, as previously reported.^3^ Patients enrolled in the primary prevention cohort in DA VINCI were categorised as per ESC/EAS risk categories (low, moderate, high or very high CV risk) based on conditions such as diabetes, FH and reduced glomerular filtration rate. These conditions were pre-existing and recorded prior to LLT. For primary prevention patients who were not captured in the aforementioned groups, SCORE risk was calculated using total cholesterol and high-density lipoprotein-C (HDL-C). These values were captured at the time of LDL-C measurement when patients were stabilised on LLT. Patients were categorised into risk categories based on comorbidities and the prevalence of ASCVD, with the exception of primary prevention patients where total cholesterol and HDL-C were used for SCORE. The LDL-C goals for each risk category, aligning with the 2019 ESC/EAS guidelines, are shown in Supplementary Table S2. For example, among patients with established ASCVD who experienced recurrent events, an LDL-C goal of less than 1.0 mmol/L was applied.

In the treatment optimisation algorithm, step 1 was the optimisation of statin therapy for patients receiving low- or moderate-intensity statins. Additional detail regarding statin intensity is described in Supplementary Table S3. The untreated LDL-C value was calculated based on the reduction in LDL-C levels expected with the statin that they were receiving (Supplementary Table S1). It was assumed that the statin dose could not be intensified in patients already receiving ezetimibe; therefore, the original LDL-C value was carried over to step 2 for patients receiving high-intensity statins or any statin and ezetimibe. Step 2 was the addition of ezetimibe for those patients not already receiving it, and the original LDL-C value was carried over into step 3 for patients already receiving ezetimibe. Step 3 was the addition of a PCSK9 inhibitor, in patients considered at very high CV risk (i.e. patients without ASCVD at very high risk or patients with ASCVD). For those not already receiving the maximum tolerated dose of statin or receiving ezetimibe, an alternative scenario was simulated in which statins were intensified and ezetimibe was added in a single step (deviating from the 2019 ESC/EAS guidelines; Supplementary Fig. S1).

At each step in the treatment optimisation simulation, an LDL-C value was calculated based on an LDL-C reduction simulated from a normal distribution truncated below 2% and above 98% for the highest dose of the statin currently used by the patient in step 1, for ezetimibe in step 2, and for a PCSK9 inhibitor in step 3. Patients who did not achieve their risk-based LDL-C goals with the addition of ezetimibe were considered equally likely to receive the PCSK9 inhibitors alirocumab or evolocumab. The mean and standard deviations (SD) for the normal distributions from which the LDL-C percentage reductions with statins, ezetimibe and PCSK9 inhibitors were simulated were taken from previous publications^8^^,^^9^ and are presented in Supplementary Table S1.

### Calculation of residual CV risk

For patients without ASCVD, the 10-year risk of a first CV event was predicted using the approach recommended by the 2019 ESC/EAS guidelines^1^ (i.e. to multiply SCORE estimates by 3 in men and by 4 in women). For each patient with ASCVD, the baseline risk of a subsequent CV event in the next 10 years (10-year CV risk) was predicted using the REACH equation,^10^ based on their demographics and their medical history. The 10-year risk of a subsequent CV event was calculated by converting the 20-month risk predicted from the REACH equation,^10^ assuming a constant rate over time (exponential survival function).

The RRR was simulated by randomly sampling from the inverse probability distribution of the rate ratio per 1.0 mmol/L from the Cholesterol Treatment Trialists’ Collaboration meta-analysis.^11^ The ARR in LDL-C achieved by treatment optimisation for each patient was also calculated. CV risk, RRR and ARR were also calculated for patients with CAD, PAD and CeVD.

### Statistical analysis

Baseline categorical demographics and clinical characteristics were reported as absolute and relative frequencies (*n* [%]). Simulations were performed 1000 times using SAS Enterprise Guide 8.2. The number of patients receiving each type of LLT (low-, moderate- or high-intensity statin ± ezetimibe ± PCSK9 inhibitor) and who achieved their 2019 ESC/EAS risk-based LDL-C goals was determined for each step of treatment optimisation. The proportion of patients was summarised using the mean and SD of all 1000 simulated proportions. Data were summarised separately by risk category. The LDL-C levels achieved after treatment optimisation, residual CV risk, ARR and RRR were summarised using the mean and SD. In order to represent the variability between individuals, the mean and SD were calculated using 2482 patients × 1000 simulations = 2,482,000 values.

### Role of the funding source

Amgen Europe GmbH was the DA VINCI study sponsor. Imperial College, London collected the data, with operational support from Amgen Europe GmbH. The corresponding author had full access to study data and had final responsibility for the decision to submit for publication.

## Results

### Study population

Of the 4112 patients who participated in the DA VINCI study and in whom LDL-C goal attainment could be assessed, 2482 (60.4%) did not achieve their 2019 ESC/EAS risk-based LDL-C goals^3^ and were included in the simulation. Of these, 962 (38.8%) patients did not have ASCVD and 1520 (61.2%) had ASCVD. Only 50 (3.3%) patients with ASCVD experienced recurrent CV events.

Demographics and baseline characteristics varied between patients without ASCVD and those with ASCVD (Table 1). The mean (SD) age of the study population was 66.9 (10.9) years. The mean (SD) age was similar across all risk groups, except for patients without ASCVD at low risk, who were younger (39.0 [9.6] years). Patients without ASCVD at low risk were more likely to be female than those without ASCVD at high or very high risk (63.6% [*n* = 35] vs 39.1% [*n* = 160] vs 28.4% [*n* = 21], respectively).

**Table 1:** Demographics and clinical characteristics of the study population according to their risk category at baseline.

	Patients without ASCVD					Patients with ASCVD			Overall (*N* = 2482)
	Low risk (*n* = 55)	Moderate risk (*n* = 424)	High risk (*n* = 409)	Very high risk (*n* = 74)	All (*n* = 962)	Not recurrent (*n* = 1470)	Recurrent^a^ (*n* = 50)	All (*n* = 1520)	
Sex, female	35 (63.6)	251 (59.2)	160 (39.1)	21 (28.4)	467 (48.5)	483 (32.9)	17 (34.0)	500 (32.9)	967 (39.0)
Age, years, mean (SD)	39.0 (9.6)	62.3 (9.8)	69.5 (8.2)	71.1 (9.3)	64.7 (11.7)	68.4 (10.0)	67.5 (11.7)	68.3 (10.1)	66.9 (10.9)
Ethnicity, white	52 (94.5)	417 (98.3)	406 (99.3)	74 (100.0)	949 (98.6)	1368 (93.1)	43 (86.0)	1411 (92.8)	2360 (95.1)
Smoking history									
Never	39 (70.9)	265 (62.5)	227 (55.5)	28 (37.8)	559 (58.1)	592 (40.3)	10 (20.0)	602 (39.6)	1161 (46.8)
Former	10 (18.2)	114 (26.9)	121 (29.6)	14 (18.9)	259 (26.9)	606 (41.2)	27 (54.0)	633 (41.6)	892 (35.9)
Current	6 (10.9)	45 (10.6)	61 (14.9)	32 (43.2)	144 (15.0)	267 (18.2)	13 (26.0)	280 (18.4)	424 (17.1)
Familial hypercholesterolaemia	30 (54.5)	41 (9.7)	13 (3.2)	0 (0.0)	84 (8.7)	0 (0.0)	0 (0.0)	0 (0.0)	84 (3.4)
Congestive heart failure	0 (0.0)	15 (3.5)	23 (5.6)	8 (10.8)	46 (4.8)	157 (10.7)	10 (20.0)	167 (11.0)	213 (8.6)
Left ventricular hypertrophy	0 (0.0)	31 (7.3)	41 (10.0)	19 (25.7)	91 (9.5)	171 (11.6)	6 (12.0)	177 (11.6)	268 (10.8)
Atrial fibrillation	0 (0.0)	23 (5.4)	57 (13.9)	8 (10.8)	88 (9.1)	229 (15.6)	8 (16.0)	237 (15.6)	325 (13.1)
Haemorrhagic stroke	0 (0.0)	3 (0.7)	0 (0.0)	0 (0.0)	3 (0.3)	41 (2.8)	1 (2.0)	42 (2.8)	45 (1.8)
Diabetes mellitus (type 1 and type 2)	7 (12.7)	139 (32.8)	180 (44.0)	38 (51.4)	364 (37.8)	619 (42.1)	22 (44.0)	641 (42.2)	1005 (40.5)
Hypertension	9 (16.4)	267 (63.0)	345 (84.4)	62 (83.8)	683 (71.0)	1171 (79.7)	39 (78.0)	1210 (79.6)	1893 (76.3)
Chronic kidney disease	0 (0.0)	12 (2.8)	93 (22.7)	27 (36.5)	132 (13.7)	204 (13.9)	14 (28.0)	218 (14.3)	350 (14.1)
Stage 3	0 (0.0)	3 (0.7)	64 (15.6)	7 (9.5)	74 (7.7)	111 (7.6)	7 (14.0)	118 (7.8)	192 (7.7)
Stage 4	0 (0.0)	0 (0.0)	1 (0.2)	11 (14.9)	12 (1.2)	16 (1.1)	3 (6.0)	19 (1.3)	31 (1.2)
Stage 5	0 (0.0)	0 (0.0)	0 (0.0)	7 (9.5)	7 (0.7)	8 (0.5)	2 (4.0)	10 (0.7)	17 (0.7)
Vascular bed involvement									
Coronary	0 (0.0)	2 (0.5)	3 (0.7)	2 (2.7)	7 (0.7)	542 (36.9)	31 (62.0)	573 (37.7)	580 (23.4)
Cerebrovascular	0 (0.0)	1 (0.2)	4 (1.0)	1 (1.4)	6 (0.6)	628 (42.7)	22 (44.0)	650 (42.8)	656 (26.4)
Peripheral	0 (0.0)	4 (0.9)	12 (2.9)	1 (1.4)	17 (1.8)	580 (39.5)	33 (66.0)	613 (40.3)	630 (25.4)

Data are n (%) unless stated otherwise.

ASCVD, Atherosclerotic cardiovascular disease; SD, standard deviation.

a Recurrent ASCVD was defined as patients who experience at least two cardiovascular events within 2 years.

### LLT at baseline

The LLTs received by the study population at baseline and by ASCVD subgroup are shown in Supplementary Table S4 and Supplementary Table S5, respectively. Almost all patients were receiving statins (98.5%; *n* = 1448). A small proportion of patients were receiving low-intensity statins (4.8%; *n* = 120) and the majority were receiving moderate-intensity statins (57.9%; *n* = 1437). Over one-third were receiving high-intensity statins (35.5%; *n* = 881). A higher proportion of patients with ASCVD were receiving high-intensity statins than those without ASCVD (43.4% [*n* = 659] vs 23.1% [*n* = 221], respectively). Similar proportions of patients (approximately 10%), with and without ASCVD, were receiving any ezetimibe therapy (*n* = 144 and *n* = 105 for patients with and without ASCVD, respectively).

### Simulation of treatment optimisation

**Fig. 2:**
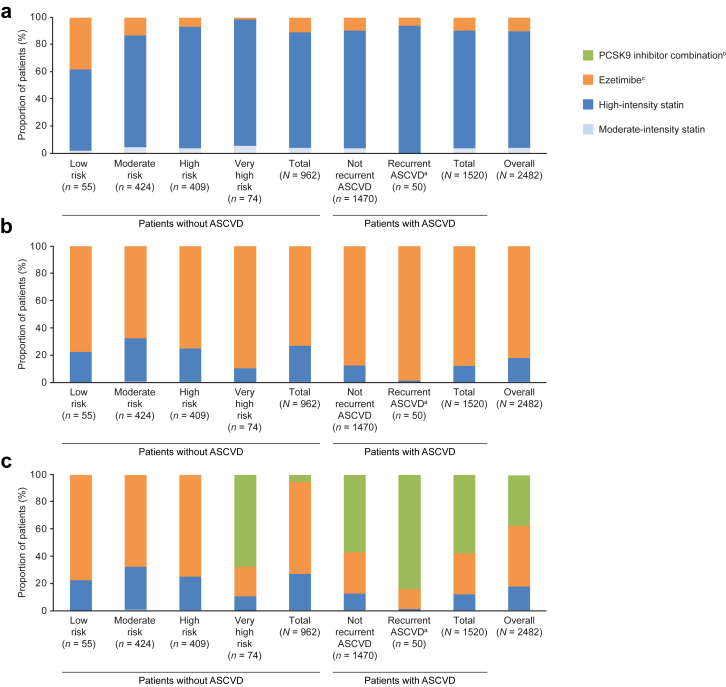
Proportion of patients receiving optimised treatment at each step in the simulation: step 1, intensification of statin therapy **(a)**; step 2, addition of ezetimibe **(b)**; and step 3, addition of a PCSK9 inhibitor **(c)**. ^a^Recurrent ASCVD was defined as patients who experience at least two cardiovascular events within 2 years. ^b^Ezetimibe includes patients on ezetimibe monotherapy or ezetimibe and statin therapy. ^c^PCSK9 inhibitor combination includes patients on any PCSK9 inhibitor therapy, including those who were initially on ezetimibe only, or on ezetimibe in combination with any statin therapy. ASCVD, atherosclerotic cardiovascular disease; PCSK9i, proprotein convertase subtilisin/kexin type 9 inhibitor.

**Fig. 3:**
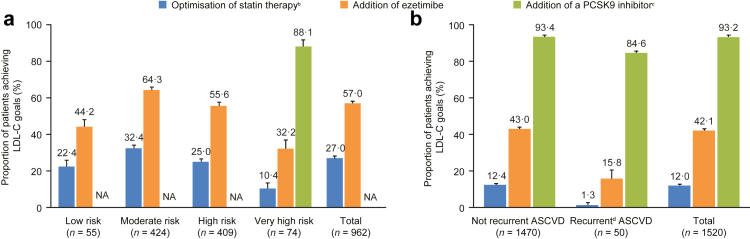
Simulation of LDL-C goal attainment through optimal implementation of the 2019 ESC/EAS dyslipidaemia guidelines^a^ in patients without ASCVD **(a)** and patients with ASCVD **(b)**. ^a^A full overview of the LDL-C goals in the 2019 ESC/EAS dyslipidaemia guidelines is detailed in Mach F et al. *Eur Heart J.* 2020; 41:111–188. ^b^Uptitration of statins for patients not already on the highest available dose of the currently prescribed statin and not already on ezetimibe. ^c^According to the 2019 ESC/EAS guidelines, PCSK9 inhibitors are only recommended for patients at very high risk. ^d^Recurrent ASCVD was defined as patients who experience at least two cardiovascular events within 2 years. Error bars show standard deviation. NA has been added to total column of patients without ASCVD, as only those at very high CV risk were eligible for a PCSK9 inhibitor per the treatment guidelines. Given that the intention of the analysis was to model the implementation of the ESC/EAS guidelines, the proportion of patients who were receiving PCSK9 inhibitors and achieved their LDL-C goals with low, moderate or high CV risk was not evaluated. ASCVD, atherosclerotic cardiovascular disease; EAS, European Atherosclerosis Society; ESC, European Society of Cardiology; LDL-C, low-density lipoprotein cholesterol; NA, not applicable; PCSK9, proprotein convertase subtilisin/kexin type 9.

The proportion of patients receiving optimised treatment at each step of the treatment optimisation algorithm is depicted in Fig. 2. The simulations of LDL-C goal attainment through optimal implementation of the 2019 ESC/EAS dyslipidaemia guidelines is shown in Fig. 3.

If statin therapy was optimised in patients without ASCVD at step 1 (*n* = 962), 85.2% (*n* = 820) of patients would be receiving high-intensity statins (Fig. 2a). This optimisation of statin therapy would result in 27.0% (*n* = 259) of patients achieving their risk-based LDL-C goals (Fig. 3a). The stepwise addition of ezetimibe would result in 61.8% (*n* = 594) of patients receiving high-intensity statins and ezetimibe (Fig. 2b), and this step would result in 57.0% (*n* = 548*)* of patients achieving their LDL-C goals (Fig. 3a). At step 3, in those at very high risk, 62.6% (*n* = 46) of patients without ASCVD would be treated with high-intensity statins, ezetimibe, and a PCSK9 inhibitor (Fig. 2c). This step would result in 88.1% (*n* = 65) of patients without ASCVD at very high risk achieving their risk-based LDL-C goals (Fig. 3a). The mean (SD) of the proportion of patients without ASCVD receiving LLT at each step is described in Supplementary Table S6.

Only 12.0% (*n* = 183) of patients with ASCVD would achieve their risk-based LDL-C goals through statin optimisation (Fig. 3b). The stepwise addition of ezetimibe would result in almost 80% (*n* = 1210) of patients receiving high-intensity statins and ezetimibe, and 42.1% (*n* = 641) would achieve their risk-based LDL-C goals. The further addition of a PCSK9 inhibitor at step 3 would result in 93.2% (*n* = 1416) of patients achieving their risk-based LDL-C goals.

Of those who had experienced recurrent CV events, only 1.3% (*n* = 1) would achieve their risk-based LDL-C goals through statin optimisation, 15.8% (*n* = 8) would achieve their risk-based LDL-C goals through the stepwise addition of ezetimibe. The addition of a PCSK9 inhibitor at step 3 would result in 84.6% (*n* = 42) of patients achieving their risk-based LDL-C goals (Fig. 3b).

### LDL-C levels

Mean (SD) LDL-C levels at baseline (i.e. before optimisation) and following simulation of the treatment optimisation algorithm are presented in Supplementary Fig. S2. Among patients without ASCVD, the mean (SD) LDL-C level was 3.1 (0.8) mmol/L at baseline and would be reduced to 2.7 (1.0) mmol/L following implementation of step 1 of the treatment optimisation algorithm (Supplementary Fig. S2). At step 2, the mean (SD) LDL-C level would decrease to 2.2 (0.9) mmol/L. Among the patients at very high-risk without ASCVD who were eligible for PCSK9 inhibitors, the mean (SD) LDL-C level after step 3 would be 1.0 (0.6) mmol/L.

Among patients with ASCVD, the mean (SD) LDL-C level was 2.3 (0.8) mmol/L at baseline and was 2.1 (0.8) mg/dL following implementation of step 1 of the treatment optimisation algorithm. The mean LDL-C levels would decrease to 1.7 (0.7) mmol/L at step 2 and to 0.9 (0.5) mmol/L at step 3 (Supplementary Fig. S2). The distribution of LDL-C levels for patients with ASCVD without recurrent events at baseline and following simulation of the treatment optimisation algorithm is presented in Supplementary Fig. S3 as an illustration. Mean (SD) LDL-C levels at baseline for the ASCVD subgroups are presented in Supplementary Fig. S4. Mean (SD) LDL-C levels at baseline in patients with PAD and CeVD were numerically higher than in patients with CAD (Supplementary Fig. S4).

### Residual CV risk

**Fig. 4:**
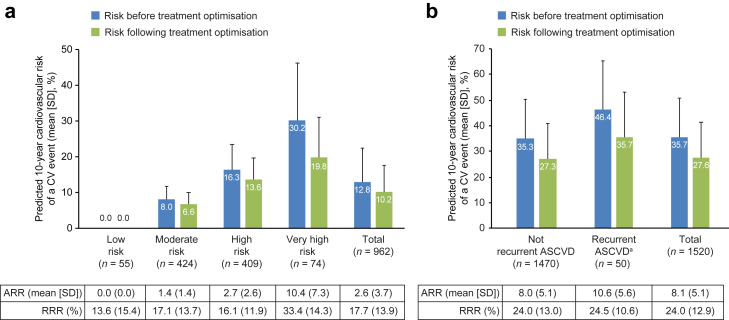
Simulated risk reduction following treatment optimisation in patients without ASCVD (SCORE) **(a)** and patients with ASCVD (REACH) **(b)**. Data are shown as mean (SD). ^a^Recurrent ASCVD was defined as patients who experience at least two cardiovascular events within 2 years. ASCVD, atherosclerotic cardiovascular disease; NA, not applicable; REACH, reduction of atherothrombosis for continued health; SCORE, systematic coronary risk evaluation SD, standard deviation.

Following optimal implementation of the 2019 ESC/EAS treatment algorithm, the absolute LDL-C risk reduction likely to be achieved was calculated and the relative risk reduction which would be achieved with this approach was simulated (Fig. 4). The residual risk of a first CV event after treatment optimisation was calculated using SCORE for patients without ASCVD. The mean (SD) predicted 10-year risk of a CV event using SCORE was 13% (10%) and 10% (7%), at baseline and after treatment optimisation, respectively (Fig. 4a). The mean (SD) simulated RRR and ARRs of a CV event using SCORE were 18% (14%) and 3% (4%), respectively (Fig. 4a).

Following simulation of the 2019 ESC/EAS treatment algorithm, the estimated risk of CV events was calculated in patients with ASCVD using the REACH equation (Fig. 4b). The mean (SD) predicted 10-year risk of a CV event using the REACH equation was 36% (15%) and 28% (14%), at baseline and after treatment optimisation, respectively. The mean (SD) simulated RRR and ARR of a CV event using the REACH equation were 24% (13%) and 8% (5%), respectively (Fig. 4b). The distribution of the risk of CV events for patients with ASCVD and without recurrent CV events, at baseline and through optimal implementation of the 2019 ESC/EAS dyslipidaemia guidelines, is presented in Supplementary Fig. S5 as an illustration. The mean (SD) predicted 10-year risk of a CV event was 32% (15%), 39% (16%) and 34% (15%) at baseline for patients with CAD, PAD and CeVD, respectively (Supplementary Fig. S6). After treatment optimisation, the mean (SD) simulated 10-year risk of a CV event was 25% (13%), 30% (15%) and 27% (13%) for the respective subgroups. The mean (SD) simulated RRR of a CV event was 23% (13%), 24% (13%) and 24% (13%) for patients with CAD, PAD and CeVD, respectively. The mean (SD) simulated ARRs of a CV event was 7% (5%), 9% (5%), and 8% (5%) for the respective subgroups (Supplementary Fig. S6).

### Alternative treatment optimisation algorithm

In the scenario where statin therapy was optimised and ezetimibe was added in a single step, 191 additional patients at very high CV risk without or with ASCVD would receive ezetimibe compared with intensifying treatment over two steps (an increase of 12.0%). Simulating this step did not provide meaningful differences in the proportion of patients who would be predicted to achieve their risk-based LDL-C goals or in the reduction in mean LDL-C levels in this scenario, compared with intensifying treatment over two steps (Supplementary Fig. S7 and Supplementary Fig. S8). A similar reduction in residual and absolute CV risk would be predicted when statin therapy was optimised, and ezetimibe was added in a single step compared with intensifying treatment over two steps (Supplementary Table S7 and Supplementary Table S8).

## Discussion

The DA VINCI study evaluated the 2016 and 2019 risk-based LDL-C goals attained among patients with and without ASCVD in Europe in routine clinical practice. Two-thirds of patients did not achieve the 2019 ESC/EAS risk-based LDL-C goals.^3^ In this simulation study, we assessed how optimisation of LLT through implementation of the 2019 ESC/EAS treatment algorithm would improve LDL-C goal attainment and reduce residual CV risk in patients who did not achieve their goals in the DA VINCI study. Results from this study suggest that over half of patients without ASCVD would attain their 2019 ESC/EAS risk-based LDL-C goals by optimising their statin therapy and adding ezetimibe. However, for patients at very high risk, including those with ASCVD, for whom the lowest LDL-C goals are recommended, the use of the combination therapy of an optimised statin, ezetimibe and a PCSK9 inhibitor would result in approximately 90% of patients achieving their risk-based LDL-C goals. Therefore, this analysis suggests that for most patients who are not currently achieving their LDL-C goals, optimisation of statin monotherapy alone will be insufficient, and we must move towards a combination therapy centric approach, in order to achieve the 2019 ESC/EAS risk-based LDL-C goals.

The stepwise addition of ezetimibe is likely to result in at least twice as many patients achieving their LDL-C goals across all risk categories, compared with statin optimisation alone. Therefore, it makes pragmatic sense to consider combination therapy with statins and ezetimibe as a first step. For those patients who fail to achieve their LDL-C goals with this step, a PCSK9 inhibitor should be considered to attain the risk-based LDL-C goals. This analysis also examined the effect of LLT optimisation on the future risk of CV events. For patients with ASCVD who are not currently at goal, if LLT was optimised as per the 2019 ESC/EAS algorithm, the estimated RRR and ARR of a CV event would be expected to be 24.0% and 8.1% over 10 years, respectively. These results have important implications for current practice and highlight the importance of optimising LLT to mitigate future CV events.

If the population mean LDL-C level before treatment is approximately between 3.0 and 3.5 mmol/L,^12^^,^^13^ patients considered to be at very high risk and patients considered to be at very high risk and with recurrent CV events are unlikely to achieve LDL-C levels below 1.4 mmol/L and below 1.0 mmol/L, respectively, without the use of combination therapy. This is analogous to the approach of managing hypertension, where patients are routinely recommended to initiate treatment with combination therapy as a first step.^14^ Examination of treatment patterns among patients newly initiated on statins but who had yet to achieve their LDL-C goals showed that the mean (SD) time-to-first treatment change was 94 (92) days and the mean time-to-second treatment change was 178 (78) days.^15^ Hence, the time required to implement the multi-step ESC/EAS treatment algorithm, which advocates a window of observation of at least 6–8 weeks for monitoring lipids after treatment initiation or intensification,^1^ is unlikely to be replicated in the real world. This means it could take a year, or longer, before the multi-step approach of adding statins, ezetimibe and a PCSK9 inhibitor will result in LDL-C goal attainment for individual patients. In the first year of treatment with LLT, CV risk is reduced by half for every 1.0 mmol/L decrease of LDL-C levels, in subsequent years the magnitude of improvement is greatly reduced. Thus, for those at highest CV risk such as patients with acute coronary syndromes, the current stepwise approach is detrimental to population health.

An alternative treatment scenario, where high-intensity statins and ezetimibe were added as a single step, was simulated in patients at very high CV risk, deviating from the 2019 ESC/EAS treatment guidelines. Using this approach, the proportion of patients at very high CV risk who would receive ezetimibe would increase by 12% (i.e. the proportion who would achieve their LDL-C goals with high-intensity statins alone). In this scenario, the same proportion of patients would attain very high-risk LDL-C goals as those who would have received high-intensity statins and then ezetimibe in a stepwise approach. Given that ezetimibe is generally well tolerated and is often generic, the 12% increase in the proportion of patients receiving ezetimibe, which could be viewed as unnecessary use of ezetimibe, would be offset by population gains. Even those patients achieving LDL-C goals on statins therapy would have a further reduction in LDL-C levels with additional estimated CV benefits. This observation provides a strong rationale for patients being treated with both high-intensity statins and ezetimibe at the same time, ensuring that they achieve their goals more quickly than through the implementation of a stepwise approach.

The use of ezetimibe as combination therapy observed in the present study is low and remains moderate, even though ezetimibe use in Europe has increased in recent years. In the recently published SANTORINI study, a real-world observational study which recruited over 9000 patients from 14 European countries between 2020 and 2021, approximately 20% of patients were receiving statins and ezetimibe.^16^ The reasons behind this are unclear. In general, this treatment is more often used as a combination therapy in patients with FH: approximately 40–70% of patients with FH may be receiving this therapy.^17^, ^18^, ^19^ In part, this may reflect the accepted paradigm that patients with FH are often managed by lipid specialists and have very high LDL-C levels and are therefore more likely to require combination therapy. A similar scenario exists for patients with statin intolerance, where there are few alternatives to statins and physicians may more readily initiate ezetimibe monotherapy in patients with statin intolerance than in those who are not statin intolerant. As post-statin treatment LDL-C levels remain high in patients receiving statins, achieving a further 20%–25% lowering of LDL-C levels with ezetimibe translates into a greater absolute lowering of LDL-C levels. Given these observations, the low use of ezetimibe is perplexing and could reflect a lack of perceived benefit from the treatment based on additional percentage reduction in LDL-C. It could also reflect a lack of awareness and of that even modest absolute LDL-C over time results in greater relative benefits over large time-horizons (cumulative exposure).^20^ Furthermore, availability of this therapy and a lack of guidance in its use and place in local care pathways may be barriers to its use. Hence it is important to positively reinforce for the physician the importance or effectiveness which is related to absolute lowering of LDL-C levels and greater absolute benefits in those at very high CV risk through the addition of ezetimibe in such patients.

The utilisation of PCSK9 inhibitors as part of combination therapy was also low in this study. This may be because combination therapies with PCSK9 inhibitors have not yet been widely adopted for several reasons, such as limitations in reimbursement policies and pricing.^21^ It has been reported that the reimbursement thresholds for PCSK9 inhibitors in most countries are much more restrictive than those recommended in clinical guidelines.^22^ Thus, lowering the LDL-C threshold for PCSK9 inhibitors reimbursement would permit more patients to receive combination therapy, increasing the likelihood of patients at highest CV risk achieving their ESC/EAS LDL-C goals.^22^ However, in addition to effectiveness, the use of PCSK9 inhibitors is also governed by cost and the guidelines recommend a highest-risk-highest-benefit approach for the use of PCSK9 inhibitors.^1^^,^^23^ It should be highlighted that the use of combination therapy with PCSK9 inhibitors results in a significant reduction in the risk of CV events. For example, in the FOURIER open-label extension study, long-term LDL-C lowering with evolocumab was associated with persistently low rates of adverse events for over eight years and led to further reductions in CV events compared with delayed treatment initiation.^24^ Therefore, the costs of PCSK9 inhibitors might be offset by the potential long-term cost savings for the healthcare system by reductions in direct and indirect costs related to CV events prevented.

Indeed, cost-effectiveness considerations of PCSK9 inhibitors have been analysed in multiple publications with varying results, and have indicated that monoclonal antibodies are only cost-effective in specific patient groups with high to very high CV risk.^23^^,^^25^^,^^26^ Interpretation of these results should take into account that cost-effectiveness is assessed differently depending on a number of factors. These include the setting, medication costs and the multiple assumptions that the model is based on. Assumptions considered can include the target population, the duration of prediction period, the estimated reduction in LDL-C levels, the frequency of predicted CV events and the impact of assumed CV events on quality of life and health care burden. In the most recent publications examining cost-effectiveness of PCSK9 inhibitors, evolocumab use was evaluated using a Markov model based on Swedish observational data from the FOURIER trial.^25^ It was demonstrated that the cost-effectiveness of PCSK9 inhibition was improved in patients with a recent history of myocardial infarction and high LDL-C levels, which was consistent with the 2019 ESC/EAS guidelines and previous data.^25^ However, it should be highlighted that economic analyses such as these are country-specific and the results may not be extrapolated to other countries. Importantly, net prices are confidential, pricing may also change over time and new economic models will then be required.

The present analysis suggests that the use of combinations of oral therapies may enable many patients to achieve their risk-based LDL-C goals. However, it should be noted that this approach relies mainly on treatments that require daily dosing. Thus, this predicates the importance of the physician reinforcing optimal adherence of a treatment to the patient to achieve the desired LDL-C reduction. The consequences at a population level of non-adherence to small molecules could be considerable. For instance, a graded relationship for adherence to LLT and CV outcomes has been described, where a 10% decline in adherence increases the relative risk of CV events by 5% compared with optimal adherence.^27^ Furthermore, among patients with the highest CV risk, a significantly lower risk of recurrent myocardial infarction and all-cause mortality was observed among those with adherence above 80% (as assessed by the proportion of days covered).^28^ However, persistence with PCSK9 inhibitor therapy is reported to be high,^29^ and a recent real-world study found that persistence with PCSK9 inhibitor therapy was over 90% after 30 months of follow-up.^30^

Although we conducted a large study involving 128 sites and 18 countries, it could be argued that the observations reported here represent a non-representative sample of patients from a self-selected group of sites, willing to participate in clinical studies. However, our findings are broadly consistent with results from unselected national records, such as the secondary prevention group in the SWEDEHEART registry of 25,466 adults with a recent myocardial infarction.^6^ The SWEDEHEART study found that simulating the 2019 ESC/EAS algorithms for LLT may lead to goal attainment in 90% of patients. In order to achieve this overall result, half of the patients would require treatment with a combination of a high-intensity statin, ezetimibe and a PCSK9 inhibitor; 29% would require treatment with a combination of a high-intensity statin and ezetimibe; and 20% would require high-intensity statin monotherapy.^6^ Our study extends and builds on these findings from a single country to a broader European population; this study also includes data on simulated CV risk, and includes patients with CAD, PAD and CeVD, as well as patients without ASCVD.

The strengths and limitations of our study merit consideration. A strength of our study is that estimates were used to simulate therapeutic reductions in LDL-C levels based on multiple clinical trials. As our source data are derived from a multinational study, our approach provides estimates that may be transferable to other European populations. In addition, rather than using an average treatment effect, a distribution was used in this analysis, with the mean and SDs obtained from previous publications.^8^^,^^9^ Therefore, the distribution reflects variation between patients within clinical trial settings; however, treatment compliance may be higher in these clinical trials than in a real-world setting. The assumptions used, therefore, may not be applicable to every individual, where imperfect adherence or compliance were not considered. It was also assumed that all patients not already receiving a high-intensity statin or ezetimibe could tolerate their statin dose being titrated to the highest available dose. However, some patients may already be receiving their maximum tolerable dose of statin. It should also be noted that the study population included patients from DA VINCI who had not achieved their LDL-C goals (66.5%). Therefore, the results, and the implications of these results, do not take into account the proportion of patients who had already achieved their LDL-C goals (33.5%). An additional limitation of this study is that it does not include novel LLTs such as bempedoic acid or inclisiran, as this analysis only simulated treatments included in the current treatment guidelines. Notably, in a recent meta-analysis which compared PCSK9 inhibitors and LLTs such as bempedoic acid and inclisiran, PCSK9 inhibitors were found to be the most efficacious non-statin LLT regimen.^8^

In conclusion, the 2019 ESC/EAS recommended risk-based LDL-C goals are likely to be attainable in over half of patients without ASCVD by optimising statins and adding ezetimibe. The majority of patients at very high risk are likely to achieve their risk-based LDL-C goals through optimisation of statins and the addition of ezetimibe and a PCSK9 inhibitor, leading to a reduction in CV risk. Thus, implementation of ESC/EAS guidelines could be simplified from 3 steps to 2, for those at highest CV risk, through the use of high-intensity statins and ezetimibe as a first step and the addition of PCSK9 inhibitors as the subsequent step where needed.

## Contributors

JB, SB, GV, NP and KKR contributed substantially to the study design and concept; NP and KKR were involved in the data acquisition; SB and GV conducted the data analyses; and all authors assisted with interpretation of the data. All authors were involved in drafting of the manuscript, provided critical revisions for important intellectual content, approved the final version submitted for publication and agreed to be accountable for all aspects of the work.

## Data sharing statement

Qualified researchers may request data from Amgen clinical trials and observational studies. Further information can be found at: https://wwwext.amgen.com/about/how-we-operate/policies-practices-and-disclosures/ethical-research/clinical-data-transparency-practices/clinical-trial-data-sharing-request.

## Declaration of interests

JB has received speaker fees from Amgen and research grant support from AstraZeneca. SB is an employee of Amgen Ltd and holds stock in Amgen. GV is an employee of Amgen (Europe) GmbH and holds stock in Amgen. ALC has received research grant(s)/support from Amgen, Eli Lilly, Menarini, Mylan, Sanofi, and Sanofi-Regeneron; and has served as a consultant for or received fees from Aegerion, Akcea, Amgen, Amryt, AstraZeneca, Daiichi Sankyo, Esperion, Genzyme, Ionis Pharmaceuticals, Kowa, Medco, Menarini, MSD, Mylan, Novartis, Recordati, Regeneron, and Sanofi. NP has received consultancy fees and financial support for research projects from Amgen and Pfizer, and financial support for arranging and speaking at educational meetings from Amgen, MSD and Pfizer. He holds no stocks and shares in any such companies. NP is supported by the National Institute for Health Research Senior Investigator Awards, Biomedical Research Centre funding, and the British Heart Foundation Research Centre Excellence Award. He has received financial support from several pharmaceutical companies which manufacture lipid lowering agents, for consultancy fees (Pfizer and Amgen), research projects and staff (Pfizer and Amgen) and for arranging and speaking at educational meetings (MSD, Amgen and Pfizer). He holds no stocks and shares in any such companies.

AJV-V has participated, or is currently participating, in research grants to Imperial College London from Amgen, Daiichi Sankyo, MSD, Pfizer, Regeneron, and Sanofi-Aventis; has received personal fees for consulting from Bayer and Regeneron; and has received fees for lectures from Akcea, Amgen, Mylan and Ferrer; all outside the submitted work. KKR reports grants from Amgen, Daiichi Sankyo, MSD, Pfizer, Regeneron and Sanofi; and has received personal fees from Abbott, Amarin, Amgen, AstraZeneca, Bayer, Beren Therapeutics, Biologix Pharma, Boehringer Ingelheim, Cargene, CSL Behring, CRISPR, Eli Lilly Esperion, Kowa, New Amsterdam, Novartis, Novo Nordisk, Pfizer, Regeneron, Resverlogix, Sanofi, Silence Therapeutics, SCRIBE Therapeutics, Vaxxinity and Viatris.
