# Supplementary material for: Optimal implementation of the 2019 ESC/EAS dyslipidaemia guidelines in patients with and without atherosclerotic cardiovascular disease across Europe: a simulation based on the DA VINCI study
Source: Lancet Reg Health Eur. 2023 Jun 8;31:100665. doi: 10.1016/j.lanepe.2023.100665 (PMC10398584; doi:10.1016/j.lanepe.2023.100665)
Supplement: Supplementary Figs. S1–S8 and Tables S1–S8 [file mmc1.pdf]

Supplementary Material

Supplementary methods 3

Supplementary Table 1. Mean (SD) percentage reductions in LDL-C associated with lipid-lowering therapy 3

Supplementary Table 2. The 2019 ESC/EAS guideline LDL-C goals for patients based on the risk category 4

Supplementary Table 3. Statin intensity definitions[6]. 6

Supplementary Table 5. Lipid-lowering therapy use by risk group. 7

Supplementary Table 6. Lipid-lowering therapy use by ASCVD subgroup. 8

Supplementary Table 7. Predicted 10-year cardiovascular risk following treatment optimisation (alternative scenario[a]) 10

Supplementary Table 8. Simulated risk reduction following treatment optimisation (alternative scenario[a]). 12

Supplementary Figure 1. Treatment optimisation simulation (alternative scenario[a]). 14

Supplementary Figure 2. Mean (SD) LDL-C levels at baseline and following optimal implementation of the 2019 ESC/EAS dyslipidaemia guidelines. 16

Supplementary Figure 3. Distribution of mean (SD) LDL-C levels for patients with ASCVD and without recurrent CV events[a] at baseline and through optimal implementation of the 2019 ESC/EAS dyslipidaemia guidelines 17

Supplementary Figure 4. Mean (SD) LDL-C levels at baseline of patients in the ASCVD subgroup. 18

Supplementary Figure 5. Distribution of the risk of CV events for patients with ASCVD and without recurrent CV events[a] at baseline and through optimal implementation of the 2019 ESC/EAS dyslipidaemia guidelines 19

Supplementary Figure 6. Simulated risk reduction following treatment optimisation by ASCVD subgroup (REACH). 20

Supplementary Figure 7. Simulation of LDL-C goal attainment through optimal implementation of the 2019 ESC/EAS dyslipidaemia guidelines for patients at very high risk without ASCVD (A) and patients with ASCVD at very high risk (B) (alternative scenario[a]). 21

Supplementary Figure 8. Mean (SD) LDL-C levels at baseline and following optimal implementation of the 2019 ESC/EAS dyslipidaemia guidelines (alternative scenario[a]). 22

References 23

Supplementary methods

Reasons for exclusion

In the primary DA VINCI study, 4112 patients were evaluable for goal attainment of whom 2735 (66·5%) did not achieve their 2019 ESC/EAS goals. For this analysis, 2482 patients were included, and 253 patients were excluded. Reasons for exclusion were the following: (i) patients receiving PCSK9i (*n* = 27), (ii) patients not receiving any statin or ezetimibe (*n* = 155), (iii) patients receiving multiple different statins (*n* = 20), (iv) patients with an unknown dose of statin (*n* = 51).

Supplementary Table 1. Mean (SD) percentage reductions in LDL-C associated with lipid-lowering therapy.

Lipid-lowering therapy	Dose, mg	Mean LDL-C reduction, %	SD for LDL-C reduction, %
**Statins**			
Atorvastatin	10	35·5	10·6
	20	41·4	13·5
	40	46·2	12·5
	80	50·2	13·8
Fluvastatin	20	17·0	8·0
	40	23·0	10·0
	80	26·0	9·0
Lovastatin	10	21·0	10·1
	20	24·0	11·0
	40	30·0	11·0
	60	34·5	11·7
Pitavastatin	2	39·0	14·6
	4	44·0	14·2
Pravastatin	10	20·0	11·0
	20	24·0	11·0
	40	30·0	11·2
	80	33·0	13·0
Rosuvastatin	5	38·8	13·2

	10	44·1	12·5
	20	49·5	13·3
	40	54·7	12·9
Simvastatin	5	23·0	11·0
	10	27·4	13·7
	20	33·0	10·4
	40	38·9	14·0
	80	45·0	11·7
**Ezetimibe**			
Ezetimibe	10	24·5	19·7
**PCSK9 inhibitor**			
Alirocumab	150 (biweekly)	62·7	22·7
Evolocumab	140 (biweekly)/ 420 (monthly)	64·7	26·9

The mean and SD statin LDL-C reductions data were provided by Cannon *et al*. The mean reductions data for ezetimibe, alirocumab and evolocumab were estimated from a Network Meta Analysis by Toth *et al.* and the SD data were obtained from specific clinical trials. [3-5]The mean reduction and SD data for pitavastatin were estimated from NCT00309777.

LDL-C, low-density lipoprotein cholesterol; PCSK9, proprotein convertase subtilisin/kexin type 9; SD, standard deviation.

Supplementary Table 2. The 2019 ESC/EAS guideline LDL-C goals for patients based on the risk category.

		**LDL-C goals**	
	**Risk category**	**mmol/L**	**mg/dL**
**Primary prevention**			
	Low	3	115
	Moderate	2·6	100
	High	1·8	70

	Very high	1·4	55
**Secondary prevention**			
Without recurrent CV events	Very high	1·4	55
With recurrent CV events	Very high	1	40

CV, cardiovascular; EAS, European Atherosclerosis Society; ESC, European Society of Cardiology; LDL-C, low-density lipoprotein cholesterol.

Supplementary Table 3. Statin intensity definitions[6].

	Low-intensity statin	Moderate-intensity statin	High-intensity statin
Atorvastatin	< 10 mg QD	10–< 40 mg QD	≥ 40 mg QD
Rosuvastatin	< 5 mg QD	5–< 20 mg QD	≥ 20 mg QD
Simvastatin	< 20 mg QD	20–< 80 mg QD	≥ 80 mg QD
Pravastatin	< 40 mg QD	≥ 40 mg QD	
Lovastatin	< 40 mg QD	≥ 40 mg QD	
Fluvastatin	< 80 mg QD	80 mg QD	
Pitavastatin	< 1 mg QD	1–4 mg QD	

QD, once daily.

Supplementary Table 4. Lipid-lowering therapy use by risk group.

	Patients without ASCVD					Patients with ASCVD			
Lipid-lowering therapy	**Low risk (*n* = 55)**	**Moderate risk (*n* = 424)**	**High risk (*n* = 409)**	**Very high risk (*n* = 74)**	**Total (*n* = 962)**	**Not recurrent (*n* = 1470)**	**Recurrent [a] (*n* = 50)**	**Total (*n* = 1520)**	**Overall (*N* = 2482)**
Statin monotherapy	34 (61·9)	368 (86·8)	382 (93·4)	73 (98·6)	857 (89·1)	1329 (90·4)	47 (94·0)	1376 (90·5)	2233 (90·0)
Low-intensity statin monotherapy	3 (5·5)	30 (7·1)	28 (6·8)	3 (4·1)	64 (6·7)	41 (2·8)	0 (0·0)	41 (2·7)	105 (4·2)
Moderate-intensity statin monotherapy	20 (36·4)	252 (59·4)	274 (67·0)	55 (74·3)	601 (62·5)	731 (49·7)	13 (26·0)	744 (48·9)	1345 (54·2)
High-intensity statin monotherapy	11 (20·0)	86 (20·3)	80 (19·6)	15 (20·3)	192 (20·0)	557 (37·9)	34 (68·0)	591 (38·9)	783 (31·5)
Ezetimibe monotherapy	1 (1·8)	13 (3·1)	8 (2·0)	0 (0·0)	22 (2·3)	22 (1·5)	0 (0·0)	22 (1·4)	44 (1·8)
Statin and ezetimibe	20 (36·4)	43 (10·1)	19 (4·6)	1 (1·4)	83 (8·6)	119 (8·1)	3 (6·0)	122 (8·0)	205 (8·3)
Low intensity statin + ezetimibe	0 (0·0)	4 (0·9)	5 (1·2)	0 (0·0)	9 (0·9)	6 (0·4)	0 (0·0)	6 (0·4)	15 (0·6)
Moderate-intensity statin + ezetimibe	8 (14·5)	23 (5·4)	12 (2·9)	1 (1·4)	44 (4·6)	48 (3·3)	0 (0·0)	48 (3·2)	92 (3·7)

High-intensity statin + ezetimibe	12 (21·8)	16 (3·8)	2 (0·5)	0 (0·0)	30 (3·1)	65 (4·4)	3 (6·0)	68 (4·5)	98 (3·9)
Any statin (monotherapy or combination)	54 (98·2)	411 (96·9)	401 (98·0)	74 (100·0)	940 (97·7)	1448 (98·5)	50 (100·0)	1498 (98·6)	1448 (98·5)
Low-intensity statin (monotherapy or combination)	3 (5·5)	34 (8·0)	33 (8·1)	3 (4·1)	73 (7·6)	47 (3·2)	0 (0·0)	47 (3·1)	120 (4·8)
Moderate-intensity statin (monotherapy or combination)	28 (50·9)	275 (64·9)	286 (69·9)	56 (75·7)	645 (67·0)	779 (53·0)	13 (26·0)	792 (52·1)	1437 (57·9)
High-intensity statin (monotherapy or combination)	23 (41·8)	102 (24·1)	82 (20·0)	15 (20·3)	222 (23·1)	622 (42·3)	37 (74·0)	659 (43·4)	881 (35·5)
Any ezetimibe (monotherapy or combination)	21 (38·2)	56 (13·2)	27 (6·6)	1 (1·4)	105 (10·9)	141 (9·6)	3 (6·0)	144 (9·5)	249 (10·0)

Data are presented as n (%).

[a]Recurrent ASCVD was defined as patients who experience at least two cardiovascular events within 2 years.

ASCVD, atherosclerotic cardiovascular disease.

Supplementary Table 5. Lipid-lowering therapy use by ASCVD subgroup.

	**CAD (*n* = 350)**	**PAD (*n* = 599)**	**CVD (*n* = 571)**	**Total ASCVD (*n* = 1520)**
Statin monotherapy	297 (84·9)	552 (92·2)	527 (92·2)	1376 (90·5)
Low-intensity statin monotherapy	7 (2·0)	17 (2·8)	17 (3·0)	41 (2·7)

Moderate-intensity statin monotherapy	137 (39·1)	306 (51·1)	301 (52·7)	744 (48·9)
High-intensity statin monotherapy	153 (43·7)	229 (38·2)	209 (36·6)	591 (38·9)
Ezetimibe monotherapy	4 (1·1)	9 (1·5)	9 (1·6)	22 (1·4)
Statin and ezetimibe	49 (14·0)	38 (6·3)	35 (6·1)	122 (8·0)
Low intensity statin + ezetimibe	2 (0·6)	1 (0·2)	3 (0·5)	6 (0·4)
Moderate-intensity statin + ezetimibe	20 (5·7)	16 (2·7)	12 (2·1)	48 (3·2)
High-intensity statin + ezetimibe	27 (7·7)	21 (3·5)	20 (3·5)	68 (4·5)
Any statin (monotherapy or combination)	346 (98·9)	590 (98·5)	562 (98·4)	1498 (98·6)
Any ezetimibe (monotherapy or combination)	53 (15·1)	47 (7·8)	44 (7·7)	144 (9·5)

Data are presented as n (%).

Supplementary Table 6. Mean (SD) of the proportion of patients receiving LLT at each step.

	n	Mean (SD)
Step 1, high intensity statin use in patients without ASCVD	962	85.2 (0·0)
Step 2, high intensity statin + ezetimibe use in patients without ASCVD	1520	79.6 (0·7)
Step 3, high intensity statin + ezetimibe + PCSK9i use in patients without ASCVD at very high CV risk	74	62.6 (4·6)

ASCVD, atherosclerotic cardiovascular disease; CV, cardiovascular; SD, standard deviation.

Supplementary Table 7. Predicted 10-year cardiovascular risk following treatment optimisation (alternative scenario[a]).

		**Patients without ASCVD**		**Patients with ASCVD**		
		**Very high risk (*n* = 74)**	**Total (*n* = 962)**	**Not recurrent (*n* = 1470)**	**Recurrent[b] (*n* = 50)**	**Total (*n* = 1520)**
SCORE	Baseline risk	30 (16)	13 (10)	NA	NA	NA
	Treatment optimised risk	20 (11)	10 (7)	NA	NA	NA
REACH event	Baseline risk	NA	NA	35 (15)	46 (19)	36 (15)

	Treatment optimised risk	NA	NA	27 (14)	36 (17)	27 (14)

Data are presented as mean (SD).

[a]For those not already receiving the maximum tolerated dose of statin or receiving ezetimibe, an alternative scenario was simulated in which statins were intensified and ezetimibe was added in a single step (deviating from the 2019 ESC/EAS guidelines).

[b]Recurrent ASCVD was defined as patients who experience at least two cardiovascular events within 2 years.

ASCVD, atherosclerotic cardiovascular disease; NA, not applicable; REACH, Reduction of Atherothrombosis for Continued Health; SCORE, systematic coronary risk evaluation SD, standard deviation.

Supplementary Table 8. Simulated risk reduction following treatment optimisation (alternative scenario[a]).

		Patients without ASCVD		Patients with ASCVD		
	%	**Very high risk (*n* = 74)**	**Total (*n* = 962)**	**Not recurrent (*n* = 1470)**	**Recurrent[b] (*n* = 50)**	**Total (*n* = 1520)**
SCORE	Absolute risk reduction	11 (7)	3 (4)	NA	NA	NA
	Relative risk reduction	34 (14)	18 (14)	NA	NA	NA
REACH event	Absolute risk reduction	NA	NA	8 (5)	11 (6)	8 (5)
	Relative risk reduction	NA	NA	25 (13)	24 (11)	25 (13)

Data are presented as % mean (SD).

[a]For those not already receiving the maximum tolerated dose of statin or receiving ezetimibe, an alternative scenario was simulated in which statins were intensified and ezetimibe was added in a single step (deviating from the 2019 ESC/EAS guidelines).

[b]Recurrent ASCVD was defined as patients who experience at least two cardiovascular events within 2 years.

ASCVD, atherosclerotic cardiovascular disease; NA, not applicable; REACH, REduction of Atherothrombosis for Continued Health; SCORE, systematic coronary risk evaluation; SD, standard deviation.

Supplementary Figure 1. Treatment optimisation simulation (alternative scenario[a]).

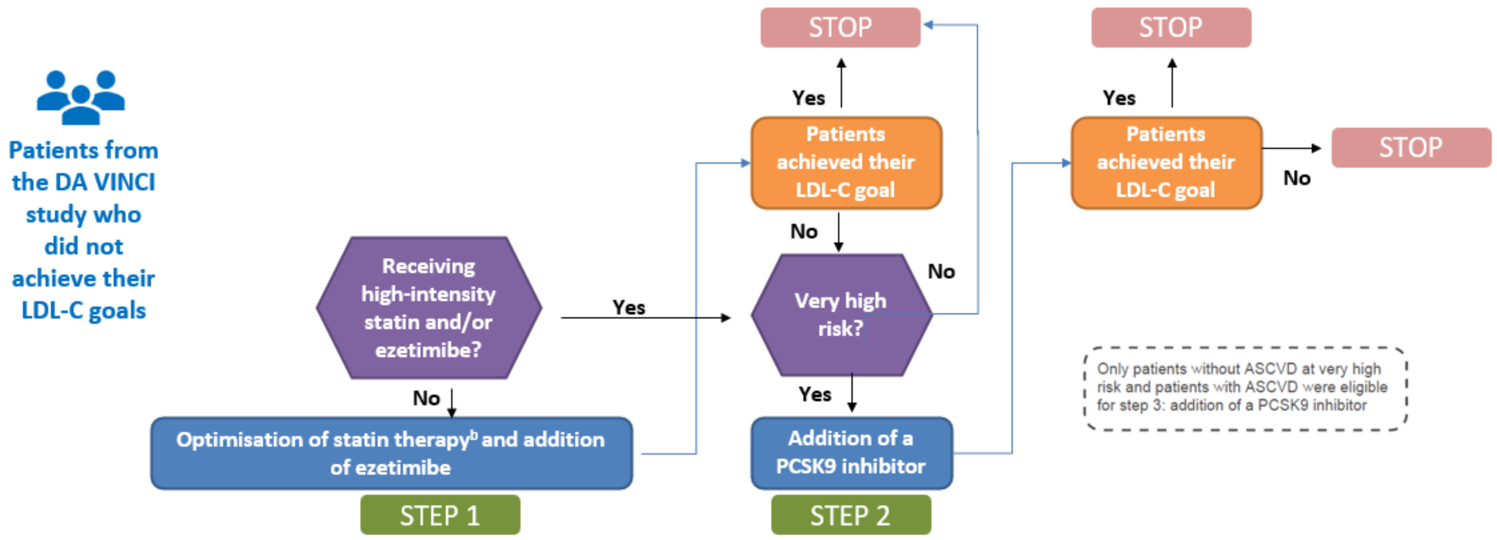

[a]For those not already receiving the maximum tolerated dose of statin or receiving ezetimibe, an alternative scenario was simulated in which statins were intensified and ezetimibe was added in a single step (deviating from the 2019 ESC/EAS guidelines).

[b]Uptitration of statins for patients not already receiving ezetimibe and not already receiving the highest available dose of their currently prescribed statin.

LDL-C, low-density lipoprotein cholesterol; PCSK9, proprotein convertase subtilisin/kexin type 9.

Supplementary Figure 2. Mean (SD) LDL-C levels at baseline and following optimal implementation of the 2019 ESC/EAS dyslipidaemia guidelines.

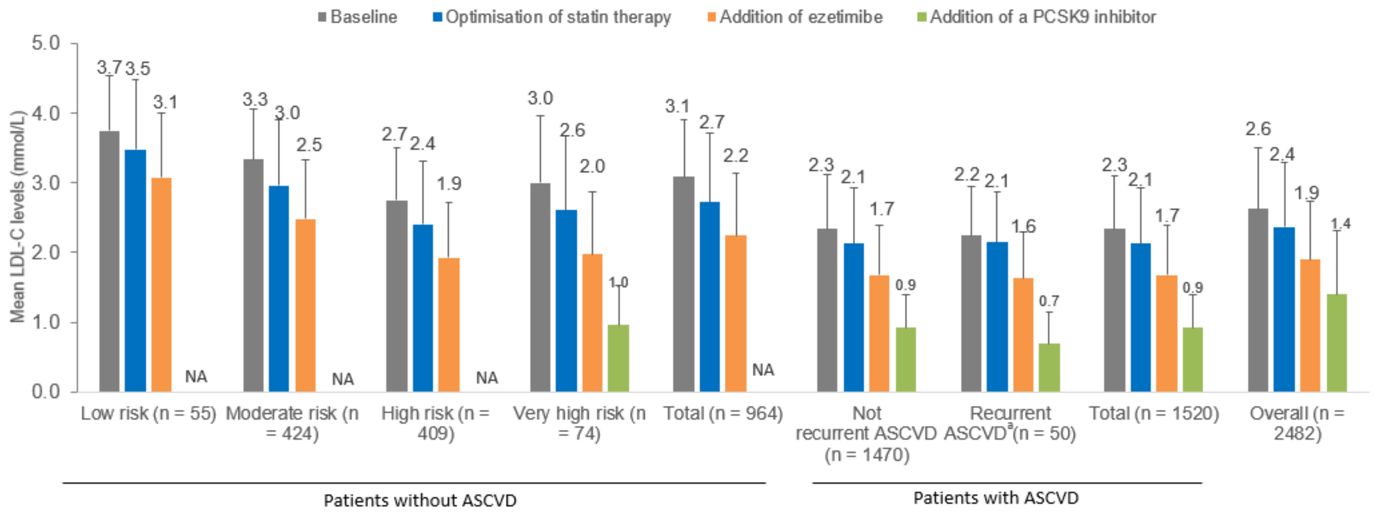

[a]Recurrent ASCVD was defined as patients who experience at least two cardiovascular events within 2 years.

ASCVD, atherosclerotic cardiovascular disease; EAS, European Atherosclerosis Society; ESC, European Society of Cardiology; LDL-C, low-density lipoprotein cholesterol; NA, not applicable; SD, standard deviation.

Supplementary Figure 3. Distribution of mean (SD) LDL-C levels for patients with ASCVD and without recurrent CV events[a] at baseline and through optimal implementation of the 2019 ESC/EAS dyslipidaemia guidelines.

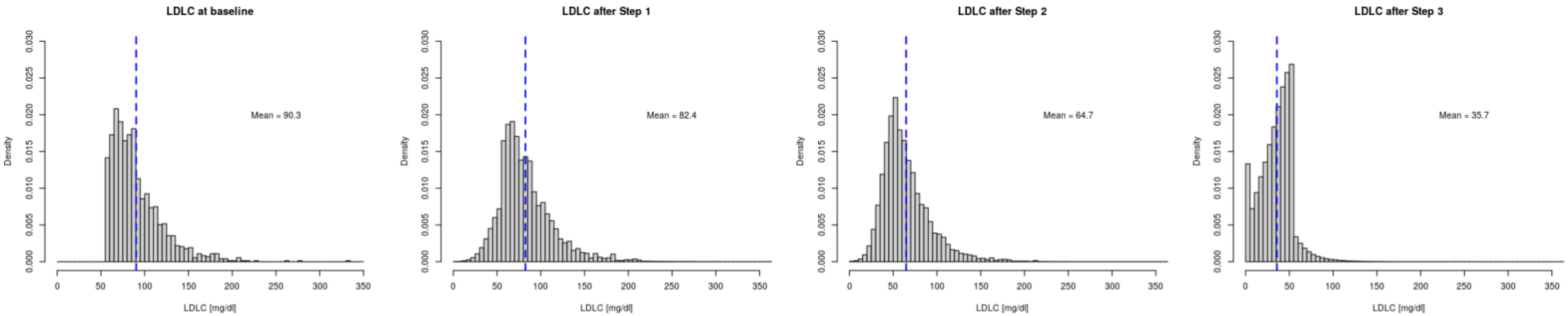

[a]The distribution for patients with ASCVD and without recurrent CV events is shown as an illustration of the simulation results. Blue dashed line represents the mean.

Supplementary Figure 4. Mean (SD) LDL-C levels at baseline of patients in the ASCVD subgroup.

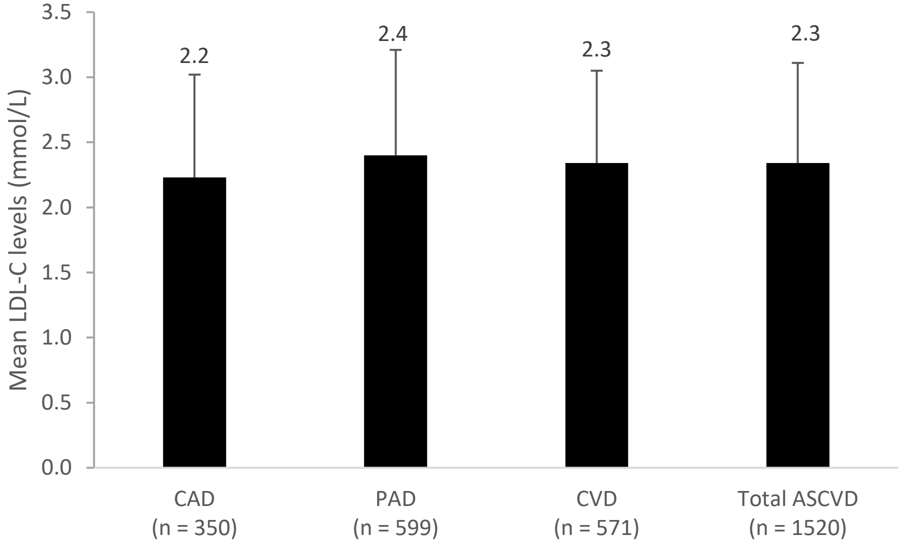

ASCVD, atherosclerotic cardiovascular disease; CAD, coronary artery disease; CVD, cerebrovascular disease PAD, peripheral artery disease; SD, standard deviation.

Supplementary Figure 5. Distribution of the risk of CV events for patients with ASCVD and without recurrent CV events[a] at baseline and through optimal implementation of the 2019 ESC/EAS dyslipidaemia guidelines.

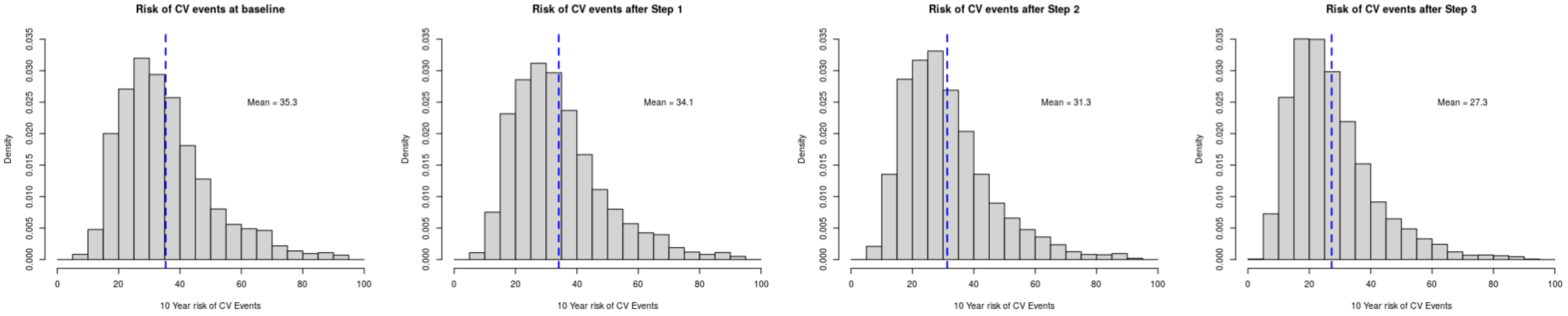

[a]The distribution for patients with ASCVD and without recurrent CV events is shown as an illustration of the simulation results. Blue dashed line represents the mean.

Supplementary Figure 6. Simulated risk reduction following treatment optimisation by ASCVD subgroup (REACH).

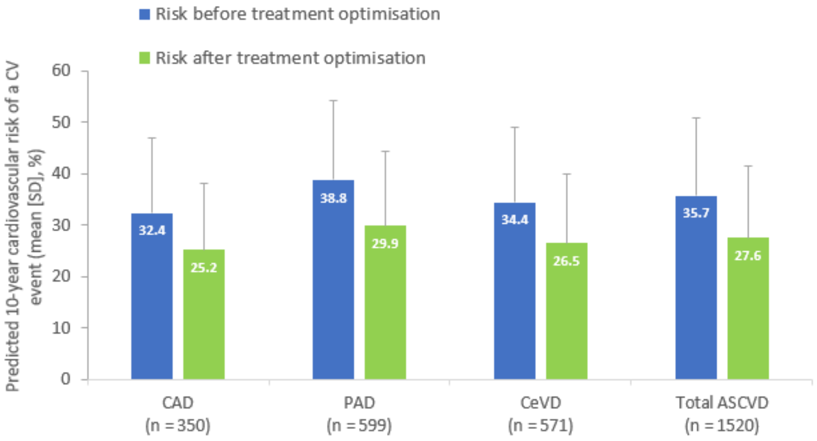

ARR (mean [SD])	7.2 (5.1)	8.9 (5.1)	7.9 (5.0)	8.1 (5.1)
RRR (%)	22.8 (12.5)	24.4 (13.3)	24.3 (12.7)	24.0 (12.9)

Data are shown as mean (SD).

ASCVD, atherosclerotic cardiovascular disease; CAD, coronary artery disease; CVD, cerebrovascular disease NA, not applicable; PAD, peripheral artery disease; REACH, Reduction of Atherothrombosis for Continued Health; SD, standard deviation.

Supplementary Figure 7. Simulation of LDL-C goal attainment through optimal implementation of the 2019 ESC/EAS dyslipidaemia guidelines for patients at very high risk without ASCVD (A) and patients with ASCVD at very high risk (B) (alternative scenario[a]).

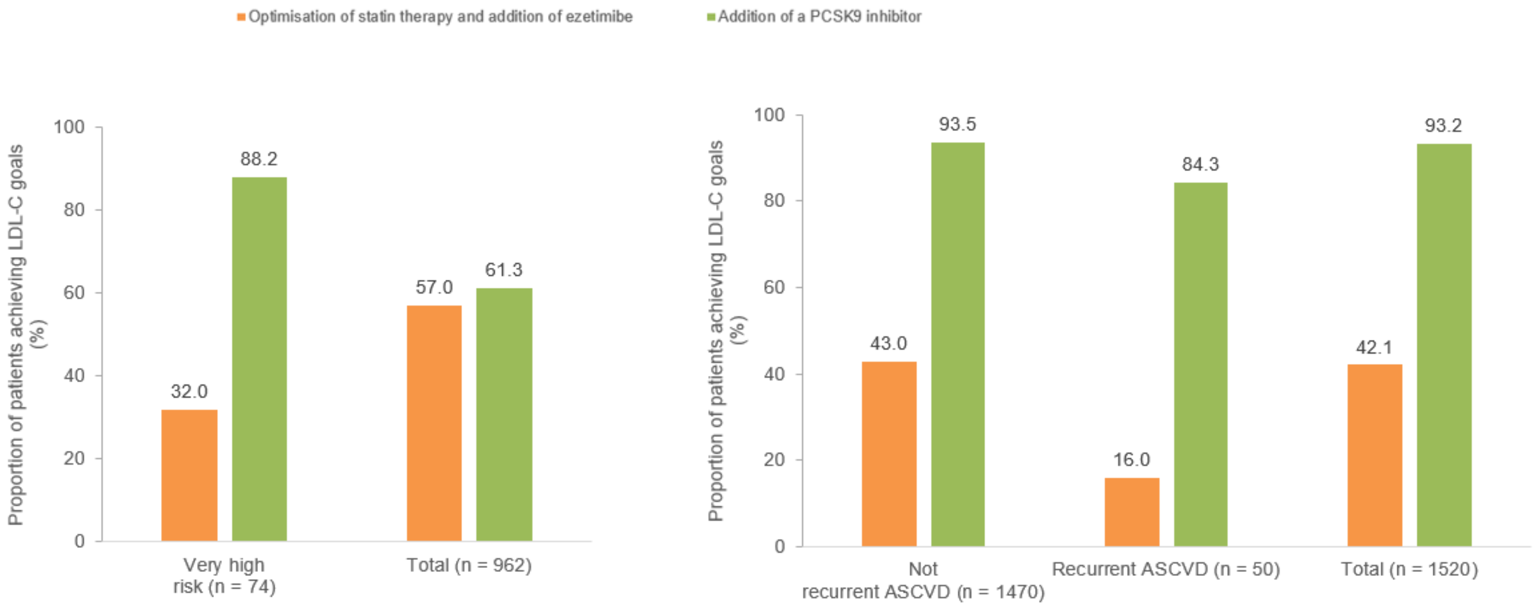

[a]For those not already receiving the maximum tolerated dose of statin or receiving ezetimibe, an alternative scenario was simulated in which statins were intensified and ezetimibe was added in a single step (deviating from the 2019 ESC/EAS guidelines).

Recurrent ASCVD was defined as patients who experience at least two cardiovascular events within 2 years.

ASCVD, atherosclerotic cardiovascular disease; EAS, European Atherosclerosis Society; ESC, European Society of Cardiology; LDL-C, low-density lipoprotein cholesterol; PCSK9, proprotein convertase subtilisin/kexin type 9.

Supplementary Figure 8. Mean (SD) LDL-C levels at baseline and following optimal implementation of the 2019 ESC/EAS dyslipidaemia guidelines (alternative scenario[a]).

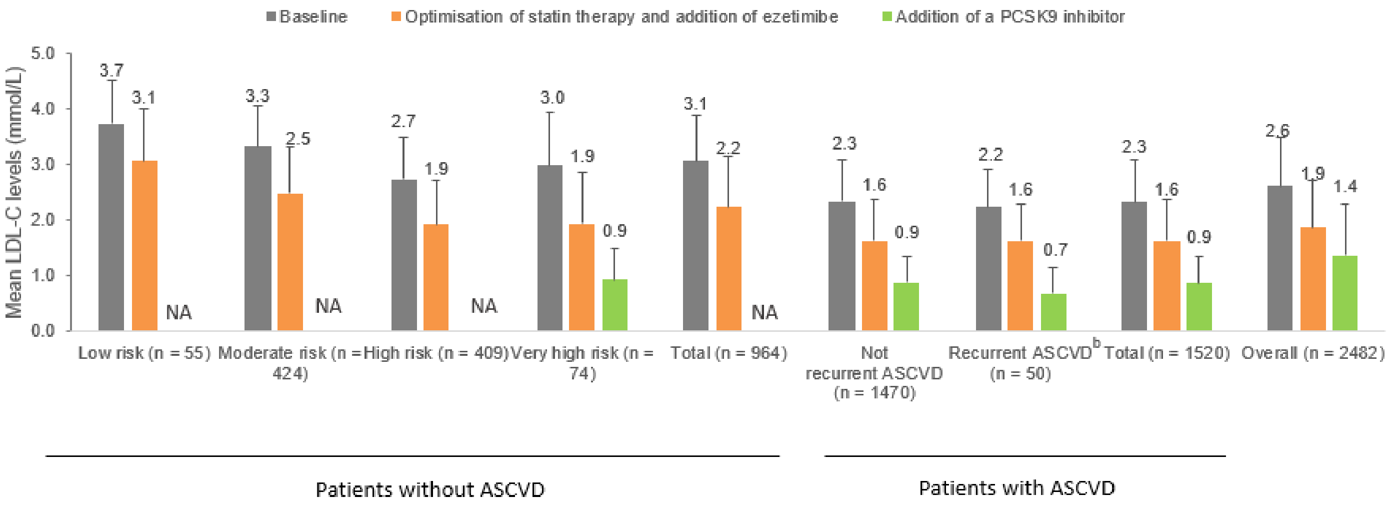

[a]For those not already receiving the maximum tolerated dose of statin or receiving ezetimibe, an alternative scenario was simulated in which statins were intensified and ezetimibe was added in a single step (deviating from the 2019 ESC/EAS guidelines).

[b]Recurrent ASCVD was defined as patients who experience at least two cardiovascular events within 2 years.

ASCVD, atherosclerotic cardiovascular disease; EAS, European Atherosclerosis Society; ESC, European Society of Cardiology; LDL-C, low-density lipoprotein cholesterol; NA, not applicable; PCSK9, proprotein convertase subtilisin/kexin type 9; SD, standard deviation.

References

1. Cannon CP, Khan I, Klimchak AC, Reynolds MR, Sanchez RJ, Sasiela WJ. Simulation of Lipid-Lowering Therapy Intensification in a Population With Atherosclerotic Cardiovascular Disease. *JAMA Cardiol* 2017; **2**(9): 959–66.
2. Toth PP, Bray S, Villa G, et al. Network Meta-Analysis of Randomized Trials Evaluating the Comparative Efficacy of Lipid-Lowering Therapies Added to Maximally Tolerated Statins for the Reduction of Low-Density Lipoprotein Cholesterol. *J Am Heart Assoc* 2022; **11**(18): e025551.
3. Robinson JG, Nedergaard BS, Rogers WJ, et al. Effect of Evolocumab or Ezetimibe Added to Moderate- or High-Intensity Statin Therapy on LDL-C Lowering in Patients With Hypercholesterolemia: The LAPLACE-2 Randomized Clinical Trial. *JAMA* 2014; **311**(18): 1870-83.
4. Robinson JG, Farnier M, Krempf M, et al. Efficacy and Safety of Alirocumab in Reducing Lipids and Cardiovascular Events. *New England Journal of Medicine* 2015; **372**(16): 1489-99.
5. Sabatine MS, Giugliano RP, Keech AC, et al. Evolocumab and Clinical Outcomes in Patients with Cardiovascular Disease. *New England Journal of Medicine* 2017; **376**(18): 1713-22.
6. Grundy SM, Stone NJ, Bailey AL, et al. 2018 AHA/ACC/AACVPR/AAPA/ABC/ACPM/ADA/AGS/APhA/ASPC/NLA/PCNA Guideline on the Management of Blood Cholesterol: A Report of the American College of Cardiology/American Heart Association Task Force on Clinical Practice Guidelines. *Circulation* 2019; **139**(25): e1082-e143.
